# Effects of Early Weaning Associated with Alimentary Stress on Emotional and Feeding Behavior of Female Adult Wistar Rats

**DOI:** 10.3390/bs12060171

**Published:** 2022-05-31

**Authors:** Víctor Isaac Meléndez Díaz, Julliet Araújo de Souza, Sandra Lopes de Sousa

**Affiliations:** 1 Graduate Program of Neuropsychiatry and Behavioral Sciences, Federal University of Pernambuco, Recife 50740-600, Brazil; julliet.jesus@gmail.com (J.A.d.S.); sandra.lsouza2@ufpe.br (S.L.d.S.); 2 Graduate Program of Nutrition, Federal University of Pernambuco, Recife 50740-600, Brazil

**Keywords:** weaning, feeding behavior, female, rats, Wistar

## Abstract

Maternal lactation proves crucial for mammals’ nutrition during their early development, influencing the development of adult physiological mechanisms. Its premature termination has been associated with several disorders, but these have been primarily documented in males, when they are most prevalent in women. Therefore, we subjected adult female Wistar rats to Early Weaning through maternal separation at age 15 days to acute alimentary stress in the form of visual and olfactory exposition to a cafeteria diet sans consumption for 22 days. We measured standard diet intake and water intake daily and cafeteria diet intake every 7 days. Additionally, we evaluated anxiety using the elevated plus maze and measured body weight in similar intervals. Results showed less consumption of the cafeteria diet among Early Weaning rats on day 2 and more time spent in the maze’s central area by the Early Weaning rats during the basal evaluation and in the maze’s open arms by control rats on day 7 when compared to the same group’s basal time. No other significant differences were found. These results show the importance of determining the impact that female steroidal gonadal hormones such as estradiol have upon feeding behavior and anxiety and determining to what degree these parameters are influenced by hormonal action.

## 1. Introduction

Knowledge of breastfeeding’s importance has evolved considerably over the last few decades. Initially dismissed as a physiological mechanism rendered irrelevant by worldwide advances in food production, sanitation, and industrialization [1], this attitude changed considerably once scientific insights revealed how much influence breastfeeding has in modulating mechanisms, both physiological [2] and otherwise [3]. Breastfeeding, or lack thereof, can influence human physiology to such a degree that infant mortality rates directly correlate with its administration [4]. Some of the mechanisms involved are those that regulate intake and food preference, with this particular pairing having important implications for adult life [5,6]. As such, there is great interest in parsing out whether breastfeeding has an influence over other behaviors that affect reward seeking.

One such behavior is anxious behavior. It is one of the most prevalent mental disorders during childhood, and its impact can extend into adulthood [7]. It is a dysfunction in an organism’s stress reactivity that is in part mediated by the hypothalamic–pituitary–adrenal axis. Both early life stress [8] and Early Weaning [9] (EW) are directly correlated with anxious-like behavior in rats. Furthermore, early life adverse conditions in rats have a direct effect on food preference [10] and food intake [11], while enhanced maternal care with increased suckling reduces anxious behavior [12]. This posits the question of how much of this effect is due to early or insufficient weaning.

Both anxiety disorders and obesity are more prevalent in women than in men. It is possible that steroidal gonadal hormones play a role in this discrepancy. However, female steroidal hormones’ effects on both types of behaviors are paradoxical, with 17β-Estradiol (E2 for short) administration having anxiolytic effects and reducing food intake in ovariectomized rats. Furthermore, female animals are oftentimes excluded from studies to eliminate the confounding effect that hormonal differences have. Therefore, there is a great need to research the interactions between environmental and nutritional stressors such as EW and their effects on a female population. As such, we set out to determine the effects of EW and an acute alimentary stressor on the feeding behavior of female Wistar rats, along with testing to see if there is an increase in their anxious behaviors.

## 2. Materials and Methods

### 2.1. Study Design

This study was a prospective, randomized, controlled, and blinded animal model study based on the Guide for the Care and Use of Laboratory Animals and the Animal Research: Reporting of In Vivo Experiments (ARRIVE) guidelines [8].

### 2.2. Animals and Experimental Protocol

#### 2.2.1. Animals

Albino Wistar rats of reproductive age approximately 200–250 g in weight were used. They were provided by the Breeding Farm of the Department of Nutrition of the Federal University of Pernambuco. Rats were paired in the ratio of two females for each male. Gestation was confirmed through weight change verification. Once gestation was confirmed, rats were transferred to individual cages and received a standard vivarium diet (Labina, Presence). After the pups’ birth, sexing was undertaken with the goal of forming litters of right pups per mother, with male and female pups being distributed per the same ratio (4:4) whenever possible. Two female pups per litter were randomly picked to form the experimental groups, each of which was comprised of eight rats. During the entire experiment, the animals were kept in standard vivarium conditions, at a 22 °C (±1) temperature, under an inverted 12 h light–dark cycle (lights on at 18 h), and with food and water ad libitum. Care and management were undertaken following the recommendations of the Brazilian College of Animal Experimentation (COBEA) and were approved by the ethics committee on animal experimentation of the Federal University of Pernambuco (UFPE) under the license number 0020/2018 on 7 April 2018.

#### 2.2.2. Early Weaning and Experimental Groups

Experimental groups were determined according to whether the animals were weaned early or not. Early weaning consisted of the early separation of pups from their mothers on a permanent basis starting from the 15th post-natal day, with control animals being weaned on the 30th post-natal day. Early-weaned pups received a shredded standard diet until 21 days of age. From the 21st day of age onwards all groups received a standard diet. Early-weaned and control groups were thus formed. After weaning, only females were used in this study. They were grouped in pairs in separate cages until experiments began at around 170 days of age. Each subgroup was composed of a total of eight animals. Therefore, the following experimental groups were made:

- Control Group—weaned on a period natural for its species (30th post-natal day, *n* = 8);
- Early Weaning Group—weaned on the 15th post-natal day (*n* = 8).

### 2.3. Behavioral Procedures

#### 2.3.1. Alimentary Stress

Alimentary stress started between 166 and 168 days of age, between 12 h and 12h20. It consisted of visual and olfactory exposure to the cafeteria diet (Bauducco chocolate cookies) for twenty minutes, over 22 consecutive days. The food was wrapped in galvanic mesh (15 cm × 15 cm) that kept animals from consuming it.

#### 2.3.2. Cafeteria Diet Intake before and after Stress

Intake measurements were carried out during five different moments: basal (sans alimentary stress) and on the 2nd, 8th, 15th, and 22nd days of alimentary stress. During basal intake measurement, animals were not exposed previously to cafeteria food wrapped in galvanic mesh; they had 1 h of free access to it. During stress days, animals had access to the cookies for 1 h after 20 min of alimentary stress. Food intake (g) was quantified through the difference between the amount offered and the remaining amount. During this experiment, the animals had free access to water.

#### 2.3.3. Body Weight

Body weight was evaluated at the start of the experiment and after that, on a weekly basis, on days 2, 8, 15 and 22 of alimentary stress. A digital weighing balance with a sensibility of 0.001 g was used.

#### 2.3.4. Experimental Anxiety before and after Alimentary Stress

Anxiety assessment was carried out using the elevated plus maze model. This test model lasts for 5 min and is carried out inside a maze, which consists of two open arms (50 × 10 cm) and two closed ones (50 × 10 × 40 cm) that extend from a central platform (10 × 10 cm), with everything at a height of 50 cm above ground level. The coating of the floor, walls, and central region is a dark anti-slip material. During the test, the maze was illuminated using low voltage red light (15 W). This experimental model was based on rodents’ innate fear of open elevated spaces and preference for closed arms. The test itself consisted of placing the animals individually on the device’s central space, with the head facing towards the open arm opposite from the evaluator and registering data during the following 5 min. Time spent in and number of entries into each arm were registered per protocol. An entry was only registered once an animal placed all four paws in one of the maze’s arms. Each animal’s time was measured using a digital chronometer. Time spent in the central platform was calculated using the following formula: TC= TT − (TBF + TBA), where TC is the time spent in the central platform, TT is the test’s total time, TBF is time spent in the closed arm and TBA is time spent in the open arm.

Using the elevated plus maze test, experimental anxiety has a basal assessment, with stress response being later assessed in four distinct periods during the 22 days of alimentary stress, these being days 1, 7, 14, and 21. Animals were tested for 5 min after being subjected to 20 min of alimentary stress during these days. The testing period went on from 12h00 to 15h00.

#### 2.3.5. Standard Diet Intake after Alimentary Stress

During the 22 days of alimentary stress, standard diet intake was measured every 24 h. A measurement of 50 g of standard diet was offered once the 20 min alimentary stress period was over. After 24 h, the remaining food was weighed. This procedure was repeated until the alimentary stress period’s end, meaning until the 22nd day. Food intake (g) was quantified as the difference between the amount offered and the remaining amount.

#### 2.3.6. Water Intake after Alimentary Stress

During the 22 days of alimentary stress, water intake was measured every 24 h. Bottles full of water weighing approximately 900 g were offered. They were weighed once the 20-min alimentary stress period was over and weighed once again after 24 h. This procedure was repeated until the alimentary stress period’s end, meaning until the 22nd day. Water intake was quantified as the difference between amount offered and remaining amount in terms of grams of weight, equivalent to milliliters of volume.

### 2.4. Statistical Analysis

Data were presented as median and standard deviations. Clinical analyses were performed by a two-way analysis of variance (ANOVA) and Test-t. The significance value used in every assessment was *p* < 0.05. Statistical analyses were carried out using GraphPad Prism 5 (GraphPad Inc., La Jolla, CA, USA).

## 3. Results

### 3.1. Body Weight

**Figure 1:**
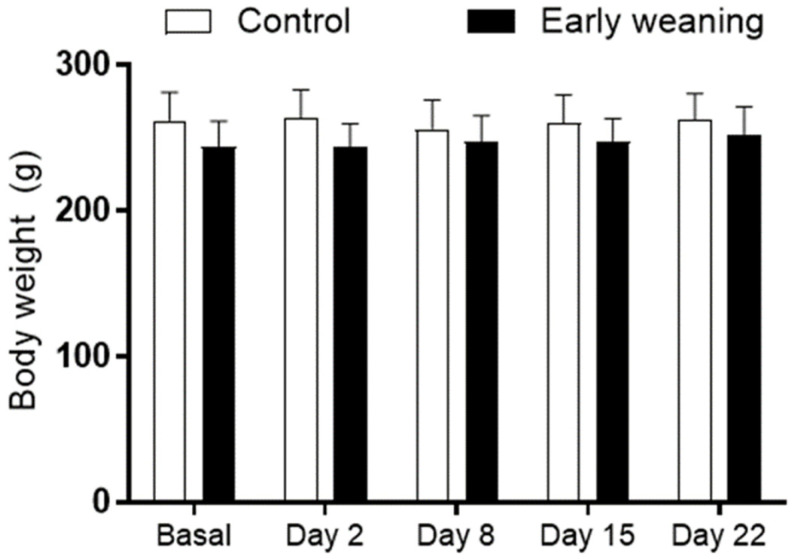
Effect of Early Weaning associated with chronic stress on body weight of adult rats. The animals’ body weight was obtained at ages 166–168 days (basal analysis) and on days 2 (169–171 days of age), 8 (175–177 days of age), 15 (182–184 days of age), and 22 (188–190 days of age) of alimentary stress. Two-way ANOVA and Bonferroni’s post-hoc test *n* = 8. Data expressed as mean ± SD.

There were no differences in body weight between the control and EW groups at any of the ages assessed (Figure 1).

### 3.2. Elevated plus Maze

**Figure 2:**
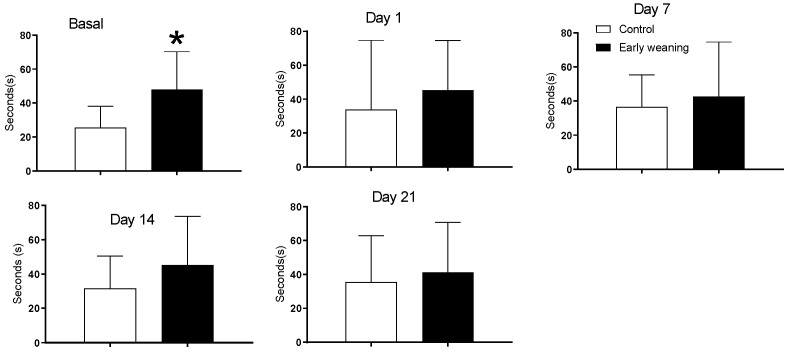
Effect of Early Weaning associated to chronic stress over time spent in central platform of the elevated plus maze. Data were obtained from animals during their 166–168 days of age (basal analysis) and during days 1 (168–170 days of age), 7 (174–176 days of age), 14 (181–183 days of age) and 21 (187–189 days of age) of alimentary stress. *t*-test *n* = 8. Data expressed as mean ± SD. * *p* < 0.05 vs. controls.

There were no differences in time spent in all four arms between the control and early-weaned group. However, an analysis of the time spent in the maze’s central platform showed that weaned animals spent more time there in the day of basal analyses, before they were exposed to alimentary stress. (Control: 25.5 ± 12.7 vs. Weaned: 47.9 ± 22.4; *p* < 0.05) (Figure 2).

**Figure 3:**
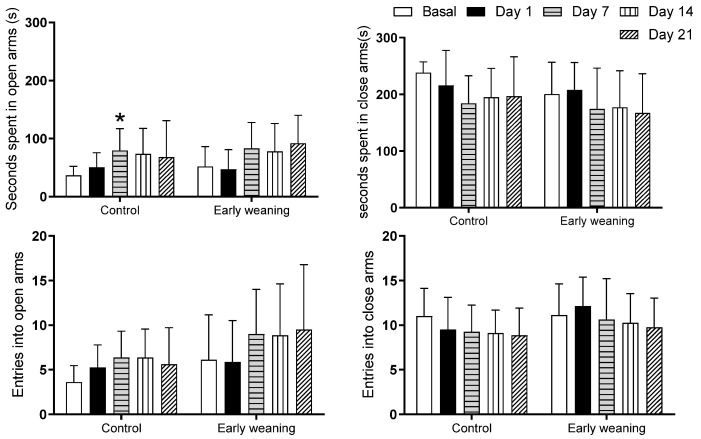
Effect of Early Weaning associated with chronic stress over time spent in open and closed arms (two upper figures) and over number of entries into open and closed arms (two lower figures) of the elevated plus maze. Data were obtained from animals during their 166–168 days of age (basal analysis) and on days 1 (168–170 days of age), 7 (174–176 days of age), 14 (181–183 days of age), and 21 (187–189 days of age) of alimentary stress. Two-way ANOVA and Bonferroni’s post hoc test *n* = 8. Data expressed as mean ± SD. * *p* < 0.05 vs. Basal.

An analysis comparing different days showed that the control group spent more time in the open arms on the 7th day than compared to the basal analysis (Basal: 36.5 ± 15.5 vs. Day 7: 79.2 ± 38.0; *p* < 0.05) (Figure 3 top). There were no differences in the number of entries into each of the four arms between the control group and the weaned group (Figure 3 bottom).

### 3.3. Cafeteria Diet Intake

**Figure 4:**
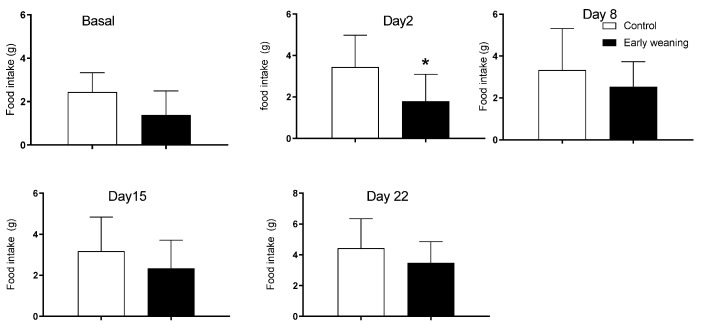
Effect of Early Weaning associated with chronic stress on chocolate cookie (Bauducco) intake. Data were obtained from animals during their 166–168 days of age (basal analysis) and on days 2 (169–171 days of age), 8 (175–177 days of age), 15 (182–184 days of age), and 22 (188–190 days of age) of alimentary stress. *t*-test *n* = 8. Data expressed as mean ± SD. * *p* < 0.05 vs. controls.

The analysis of chocolate cookie (Bauducco) intake showed an increase in the intake of the weaned group on the first day after alimentary stress started (control: 3.4 ± 1.5 vs. weaned: 1.8 ± 1.3; *p* < 0.05) (Figure 4). There were no differences in intake between groups during basal analyses nor on day 7 of alimentary stress.

### 3.4. Standard Diet Intake

**Figure 5:**
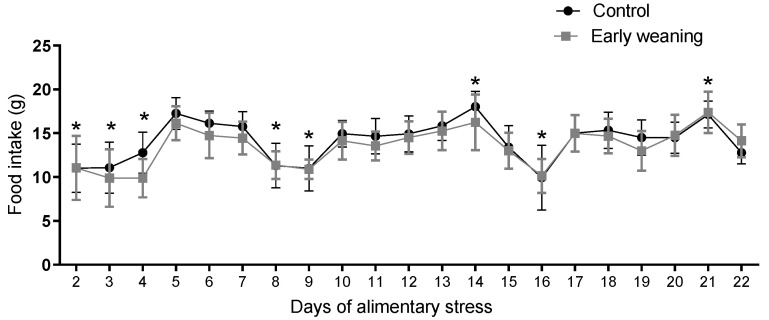
Effect of Early Weaning associated with chronic stress over standard diet intake. Data were obtained every 24 hrs between days 2 and 22 of alimentary stress. Two-way ANOVA. Bonferroni’s post-hoc test *n* = 8. Data expressed as mean ± SD. * Shows the points where both groups showed statistically lower values when compared to the highest levels.

There were no differences in standard diet intake between the control and weaned groups on any of the days assessed. However, when an analysis of the data from all the days was carried out, a similar pattern emerged in both groups, where 2 days of lower intake were followed by 5 days with higher intake during the second and third weeks of the 22 days of alimentary stress, as pictured in Figure 5.

### 3.5. Water Intake

**Figure 6:**
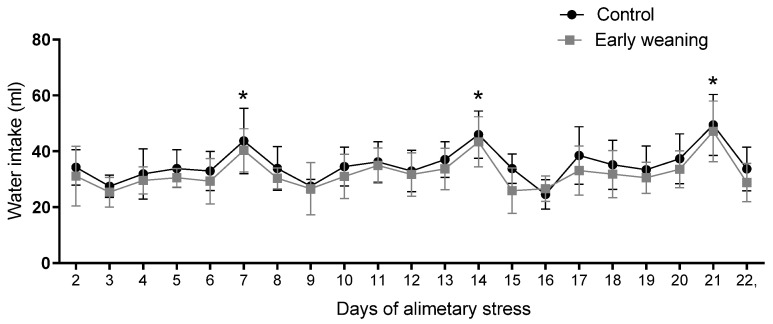
Effect of Early Weaning associated with chronic stress over water intake. Data were obtained every 24 hrs between days 2 and 7 of alimentary stress. Two-way ANOVA and Bonferroni’s post-hoc test *n* = 8. Data expressed as mean ± SD. * Shows the points at which both groups showed statistically higher values when compared to the lowest values, indicating peaks.

There was no difference in water intake between the control and weaned groups during any of the days assessed. However, when an analysis of the data from all the days was carried out, a similar pattern emerged in both groups. Intake peaked every 7 days during the 22 days of alimentary stress, as pictured in Figure 6.

## 4. Discussion

In the present study, we assessed the feeding and anxious patterns of female Wistar rats subjected to Early Weaning. Compared to control females, they showed no differences in body weight, in an anxiety test after basal assessments, and in standard diet and water intake. There was only a difference in cafeteria diet intake on the second day of acute alimentary stress.

Early Weaning (EW) is a procedure that allows us to evaluate the impact that the absence of maternal milk has on diet and maternal care during a critical moment of a mammal’s development: the neonatal period. In male rats, EW initially causes weight loss during the neonatal period; however, this result reverts during adulthood [9]. This effect is in part mediated by the metabolic programming of leptin, with low levels during infancy conditioning leptin resistance during adulthood [9]. Leptin acts on a variety of neuroanatomical structures, chief among them the hypothalamus’s archeate nucleus, where it inhibits NPY/AgRp/GABA neurons through ATP-sensitive potassium channels [10].

The actions of E2 are probably responsible for the difference in results we had when compared to similar studies carried out in males. Male rats subjected to EW show increased body weight during adult age [9], along with resistance to leptin and higher levels of this hormone [9]. Curiously enough, when subjected to EW, along with alimentary stress, they do not express this increase. Male rats subjected to EW show an increased intake of both standard and cafeteria diets [11].

Estrogens are a critical factor in body weight regulation. Everything points to food intake being controlled cyclically in a manner correlated with estrogen levels [12,13]. Studies carried out with early-weaned female rats show an increase in weight in teenage rats, something that we did not observe in our study [14]. This is possibly due to serum estrogen levels varying according to the rat’s age, these being lower in younger rats [15]. Lower levels of circulating estrogen promote higher body weight, as demonstrated in ovariectomized rats [16,17]. Classic studies have demonstrated that estrogen treatment directly results in weight loss and reduced food intake in ovariectomized rats [18]. It could be that in the present study, the similarity in body weight between both groups was related to a similar lack of change in standard diet intake. Estrogen has an important role in mediating energy balance, reducing food intake, and increasing energy expenditure [19]. Early Weaning promotes weight gain in male animals but does not have a similar effect in females, probably due to estrogen’s actions in weight reduction, meaning that estrogen did not allow for EW-induced weight gain to take place, as it has an antagonistic effect on this variable. It is worth considering that apart from its effects on energy intake, estrogen also stimulates brown adipose tissue thermogenesis through its actions on the ERα receptor [20].

Intragroup standard diet intake results did, however, vary significantly between peaks of consumption that took place on days 14 and 21 and low points on days 2, 3, 4, 8, 9, 15, 26, and 22. We posit that this was a result of cafeteria diet administration on days 2, 8, 15, and 22, as cafeteria diet intake can reduce standard diet intake [21]. Despite this, standard diet intake did not differ between both groups. However, in males subjected to EW, there is an important increase in standard diet intake, especially during the dark cycle [9]. It is precisely during the dark period of the circadian cycle that females reduce meal size during estrus [14], a phase that is preceded by a peak in serum estrogen levels. Females have demonstrated markedly rhythmic behavior during the ovarian cycle [12]. Reduced food intake during estrus involves cyclical alterations in the neurobiology of meal size control [12]. Additionally, rats show low calorie efficiency [18]. In female rats’ case, leptin and estrogens interact in such a way that ovariectomized rats show high levels of leptin, along with increased body weight [22]. Leptin is an anorexigenic hormone. Among estrogens, 17β-Estradiol (E2 for short) has considerable influence over feeding [23]. It plays a role in the homeostatic control of feeding behavior by exerting both tonic [24] and phasic [25] anorexigenic effects. The former is evident in rats without ovaries, which are the main source of estrogens in females, which subsequently show an increased intake [24]. Phasic inhibition is evident through the fluctuations in intake that happen throughout the estrous cycle [25]. Along with a decrease in food intake during estrus comes a decrease in meal size. E2′s actions over feeding behavior are attributed to that hormone’s actions in the hypothalamus and nucleus tractus solitarius (NTS) [20]. Direct administration of E2 into the arcuate nucleus of the hypothalamus directly inhibits NPY/AgRP neurons, which are orexigenic. This results in reduced food intake and body weight, as well as an increase in physical activity [26]. This action is mediated by the Erα receptor, which acts both through nuclear signaling [27] and through membrane signaling in mice and guinea pigs [28].

We know that estrogens reduce food intake; however, their effects on food reward are much less clear, specifically when it comes to food’s motivational properties. Among male rats, it has been observed that EW increases cafeteria diet intake [14]. They show a higher increase of dopaminergic and serotoninergic receptors associated with higher cafeteria diet consumption [14]. However, in this study, we did not observe changes in female rats’ intake, regardless of what kind of diet they were offered, whether it was standard or cafeteria. Rats are more motivated to work for cocaine during estrus when compared to other phases of the cycle [29,30]. This is relevant, as cerebral circuitry of food motivation overlaps with that of drug addiction. [31] However, in females, it is probable that reward mechanisms for drugs and food are more distinct from each other than in males [20]. Cafeteria diet intake in females is due more to its high caloric content than to its palatable properties [32]. Estrogens decrease food intake [16], while the use of recreational drugs increases serum estrogen levels [33]. Food motivation varies along the estrous cycle, with the least amount of food motivation registered during estrus [23]. During this same experiment, the removal of the ovaries increased food-motivational behavior. It also demonstrated that estrogens act directly on the mesolimbic reward of circuits that reduce food reward, as direct E2 microinjections in the ventral tegmental area (VTA) reduced food reward behavior [23]. Additionally, E2 decreases sucrose motivation in rats [34] while not increasing the effects of food reward [35]. This is mediated by a mGluR5-dependent mechanism that selectively increases the motivation for cocaine but not for sucrose [35]. Going from there, we may conjecture that the presence of E2 bestows a protective effect against acute alimentary stress. On top of this, orexins have differing effects on sucrose intake. [36]

The instance of a significantly reduced cafeteria diet we observed among EW rats on day 2 could be explained by a higher susceptibility to stress among female EW rats that is nevertheless not strong enough to be expressed significantly in the other parameters we measured. Nevertheless, lower cafeteria diet, standard diet, and water intakes were consistently lower in the EW group relative to controls, just not in significant enough magnitude. Some studies have shown an increase in anxious behaviors among EW rodents, particularly male ones [6,37,38]. Perhaps a more sensible way of measuring the way stress affects feeding, such as the Novelty Suppressed Feeding test, which measures latency to feeding in an unfamiliar environment, could parse this out [39]. Evaluating the effect that an anxiolytic agent would have over this behavior could help clarify this possibility, too.

Within the control group, we observed a significant increase in time spent in the open arms of the EPM during day 7 when compared to the same group’s basal assesment. This is in line with a previous study carried out to measure variability in repeated EPM assesments in non-stressed male rats, where significant changes were observed towards the fourth repeated session while still remaining below a preference threshold [40]. However, there were no differences in the time spent in the open arms of the elevated plus maze between both groups. Our results are consistent with results from previous studies carried out with EW rats, where male rats spent less time in the open arms and entered them less frequently, while there were no differences in either time spent or frequency entering between both female groups [37]. Additionally, there were similarities with studies carried out in ovariectomized mice where doses of E2 had an anxiolytic effect, increasing the time spent in the open arms of the maze during the elevated plus maze test [41]. Based on that, we may conjecture that these results are the product of the anxiolytic property of estrogens, E2 being chief among them. These actions vary according to which receptor they activate. For example, studies carried out in knockout mice found that the ERα receptor regulates the hormone’s anxiogenic effect, while ERβ knockouts and agonists, such as diarylpropionitrile and WAY-200070, increase anxious symptoms in tests such as the elevated plus maze in both rats and mice. Furthermore, the GPR30 membrane receptor is involved in the stress response since it shows increased immunoreactivity in the basal amygdala in ovariectomized mice subjected to stress. This results in a decrease in time spent in the EPM’s open arms [42]. The increase in time spent in the maze’s central area that we observed among EW rats during basal testing is a curious datum that could point towards changes in unknown behavioral parameters caused by EW. Until now, time spent in that area has been correlated with increased impulsivity during operant conditioning tasks [43]. Impulsivity has been linked to anxiety-like behavior both in rats [44] and, more controversially, in humans [45].

As for water intake, we know that it is prandial in rats, with 70–85% of spontaneous intake directly related to feeding [46]. This could be the reason why there were no differences in water intake between groups during our experiment since water intake closely mirrored food intake. We did observe peaks on days 7, 14, and 21, but we believe these were caused by additional time during which both groups had access to the water bottles as they awaited behavioral testing (EPM) compared to the rest of the days. Nevertheless, E2 is also involved in the regulation of this behavior. It has an anti-dipsogenic effect, even though that effect seems to be mediated more by organizational differences since hormone injections do not have the same effect on male rats. The structures that regulate this effect are mainly concentrated in the hypothalamus. These include the Subfornical Organ, the Lateral Hypothalamus, the Paraventricular Nucleus, and the Median Preoptic Nucleus [47]. Even though water intake in rats is prandial, E2′s anti-dipsogenic effects do not result in a decrease in food intake. Central hormone injections result in a decrease in water intake in rats, especially when administered in the lateral hypothalamus, [48], while chronic subcutaneous administration increases water intake [49].

Our study shows what we believe to be a protective effect of gonadal steroidal hormones against feeding behavior dysregulation and anxiety-like symptoms among female rats subjected to Early Weaning and alimentary stress. While the results are consistent with previous literature, they appear to be contradictory with the reality of the higher prevalence of feeding and anxiety disorders among women. Our results show the importance of continued research into the complex interactions between Early Weaning, diverse diets, and hormonal states. The use of ovariectomization, specific estrogenic receptor antagonism at different ages, and anxiolytic agents could help parse out these interactions and possibly bring about new dietary interventions and pharmacological treatments to combat the illnesses that result from their imbalance.

## Data Availability

The data presented in this study are available on request from the corresponding author.

## Abbreviations

EW	Early Weaning
E2	17β-Estradiol
EPM	Elevated Plus Maze
NPY	Neuropeptide Y
AgRp	Agouti-Related Protein
GABA	γ-aminobutyric Acid
mGluR5	Metabotropic Glutamatergic Receptor 5
ERα	Estrogen Receptor Alpha
OECD	Organization for Economic Cooperation and Development
UFPE	Federal University of Pernambuco
CEUA	Ethics Committee on the Use of Animals
CAPES	Coordination for Improvement of Higher Education Personnel
CNPq	Brazilian National Council for Scientific and Technological Development
PROCAD	National Academic Cooperation Program
